# Hypersensitivity and Applications of Cladding Modes of Optical Fibers Coated with Nanoscale Metal Layers

**DOI:** 10.3390/s18051518

**Published:** 2018-05-11

**Authors:** Jacques Albert, Fu Liu, Violeta Marquez-Cruz

**Affiliations:** Department of Electronics, Carleton University, Ottawa, ON K1S 5B6, Canada; nwuliufu@163.com (F.L.); violeta.marquez@gmail.com (V.M.-C.)

**Keywords:** fiber gratings, optical fiber sensors, surface plasmons, metal nanoparticles

## Abstract

Theoretical and experimental results are presented to show that the complex effective index of the modes of optical fibers coated with non-uniform metal coatings of gold, silver, copper, or palladium, with thicknesses between 0 and 20 nm, acquire a greatly enhanced sensitivity to various forms of perturbations. Thickness changes of less than 1 nm can be measured as well as the binding of record low concentrations of chemical and biochemical species.

## 1. Introduction

Metal coated optical fibers and waveguides (the acronym OWG will be used as a generic term covering all kinds of optical waveguiding structures, to simplify the text) are widely used in chemical and biochemical sensing applications for two interrelated reasons, namely: plasmonic enhancement of the sensitivity and well established techniques to attach functional layers, and bio-recognition elements on metals, most notably gold and silver [1,2,3,4,5]. For such applications to work, the metal layers or metal particles must be thin enough or sparse enough to let some light through, so that it can interact with the measurands and result in measurable changes in the optical properties of the OWG. Apart from this basic requirement, the morphology of the metal structures is important, because the plasmonic enhancement relies on the localization of the electromagnetic energy density on the OWG. This can occur in two very distinct ways. In the case of the uniform, highly conducting thin films, surface plasmon polariton waves (i.e., waves bound to a metal-dielectric interface and propagating along it) can be excited, either by tunneling across a thin film that is bounding an interface, where a light beam is totally internally reflected, or by grating assisted coupling [1,2]. This class of devices are referred to as ‘surface plasmon resonance’ (SPR) devices. It is also possible to use end-fire coupling to launch the SPP wave from the edge of the metal layer, but this is only practical for a special class of ‘long range surface plasmon’ devices (LRSP) that are made up of very thin metal layers surrounded by materials with similar permittivity values [6], otherwise the propagation lengths of the SPP waves for any realistic metals do not exceed a fraction of millimeter [6]. In the case of layers of metal particles, the plasmonic enhancement is usually attributed to so-called ‘localized SPR’ (LSPR), whereby the incident optical waves drive electronic resonances at very specific wavelengths, which are related to the metal plasma frequency and to the sizes and shapes of the particles (or equivalently, of holes in a uniform layer) [4,7]. In LSPR, end fire coupling is impossible as these structures do not support the propagating waves. However they can be deposited onto OWG (or inside them, in the case of microstructured fibers) [8] to interact with the guided modes of the OWG. They are also widely used in bulk optic applications (including many forms of microscopy), in which case they are simply deposited onto suitable substrates and their optical properties are measured by reflection or transmission spectroscopy [7].

In both SPR and LSPR applications, the sensitivity enhancements result from the fact that the incident light energy becomes highly localized along the metal surfaces when the SPR and LSPR conditions are met. Then, regardless of the excitation configuration, a larger fraction of the interrogating light overlaps with the perturbations of the medium, when these perturbations occur in close proximity to the metal surfaces, thereby magnifying the observed spectral transmission or reflection changes.

The purpose of the study that is presented here is to examine the special cases of the ultrathin (less than 10 nm) and very sparse metal coated OWG sensor devices, because of recent observations of highly anomalous sensing results [9,10,11,12], and also because such coatings possess linear and nonlinear optical properties, which are vastly different from those of thicker, larger metal structures [13]. It will be shown that the ultrathin or sparse metal films acquire a very large form birefringence (i.e., a birefringence not as a result of an intrinsic asymmetry of the material itself, but rather to its geometry) of the order of 10%, and furthermore, that the OWG sensors made with such films have greatly enhanced the sensitivities that do not appear to be because of plasmonic resonances, an effect that we call ‘hypersensitivity’.

## 2. Materials and Methods

As exemplary OWG structures, the standard single mode fibers that were operating in the near infrared are used (Corning SMF28). These fibers were widely available and at a very low cost, as they were mass-produced for telecommunication applications. Furthermore they were ‘photosensitive’ to ultraviolet (UV) light and it was possible to fabricate short period gratings in them by irradiation with a periodic light pattern, which was created by a diffractive optical element or any other form of interference pattern [14,15]. These were called fiber Bragg gratings, or FBGs, and they were produced commercially by many vendors, for applications in communications, high power laser sources, and sensing. In our work, to study the thin metal coatings, light needed to be extracted from the core of such fibers into the cladding, where it was totally internally reflected at the boundary, with an evanescent field extending outside the fiber, which could probe the coatings on the surface. This was done through ‘tilted’ FBGs (TFBGs), which were obtained, as the name implies, by an inclination of the fiber in the UV interference pattern during the photo-inscription, which was otherwise identical to that which was used for the conventional FBG fabrication [16,17,18,19,20,21,22]. The detailed description of the TFBG fabrication and main features were described in [22], but in short, for all of the examples that were given here, the gratings were 10–15 mm long and the tilt angles ranged from 6°–10° (relative to the normal from the fiber axis). The most important aspects of TFBGs, for the purpose of the present paper, were as follows: (1) the cladding of the fiber behaved as a highly multimode structure, which supported hundreds of individual propagating modes; (2) because of the resonant nature of the coupling between the core and cladding modes, these could be excited individually by changing the wavelength of the incident light that was propagating in the core; and (3) when the input light was linearly polarized, the cladding modes with electric fields, which were polarized either radially or tangentially, to the cladding surface could be excited selectively. The measurements were carried out in several different (but equivalent) methods, namely: (1) by launching a broadband light into the core (using fiber-coupled amplified spontaneous emission sources, such as the JDSU BBS1550) and detecting transmission spectra with an optical spectrum analyzer (Ando AQ6317B); (2) with a JDSU OMNI 2 swept wavelength system, which consisted of a scanning tunable laser synchronized with a power detector; and (3) with a LUNA OVA 5000, which was also based on a tunable laser and synchronized photo-detector. The latter two systems could perform the automatic polarization diverse measurements, but in order to obtain the individual spectra with the modes that were polarized either radially or tangentially, a computer-controlled fiber-coupled polarization controller (JDSU PR2000 was inserted between the source and the TFBG).

The simulations were carried out in two steps. Firstly, the modes of the core and cladding were calculated using a cylindrical finite-difference vector mode solver, capable of handling an arbitrary number of layers with real or complex permittivities, including the effect of material dispersion [23,24,25]. Then, the transmission spectra were calculated by coupled mode theory, which was described in detail for TFBGs by Erdogan and Sipe in [20]. Using this simple approach, the effect of adding ultrathin metal coatings could be simulated and compared to the experimental results. The properties that were used for the fiber were as follows: core radius = 4.1 μm, cladding radius = 62.5 μm, cladding material of pure silica (SiO_2_), and core material of germanium-doped silica with 0.0525 germanium/silicon ratio. The refractive index for the germanium-doped core and pure silica cladding, as a function of wavelength and germanium doping, were taken from [26]. The exact doping value for the core of the fiber that we used was not known, but it could be found by fitting a simulated spectra of TFBGs in air to the experimental ones [27]. It must be pointed out that the grating photo-inscription process changed the refractive index of the core by the average of the photo-induced refractive index modulation, so the germanium doping that was used to fit the experimental spectrum was not the actual one, but rather an ersatz made up of the actual concentration with a correction that was related to the photo-induced index.

Finally, the simulation of the particulate coatings normally involved effective medium approaches (such as Maxwell-Garnett or Bruggeman models) where the resulting permittivity was obtained as a combination of the bulk permittivity of the constituents (the metal particles and surrounding dielectric), the fill factor of the metal, and with various geometric parameters [28,29]. However, for the structures with sizes of the order of 10 nm and less, and metals in particular, the bulk values no longer applied, since the lateral dimensions (thickness in the case of uniform films, if ever such films could be uniform at these thicknesses, which was unlikely [30]) were smaller than the mean free path of electrons in these metals (between 37.7 and 53.3 nm for gold, silver, and copper) [31]. This was why it was observed from many studies that such thin metal films behaved as dielectrics with quite high dielectric constants [13]. Therefore, the results that are to be presented did not assume a priori values for the permittivity of the metal coatings, and rather used modelling to find the effective permittivity that provided the best fits to the experimental observations. Furthermore, since TFBGs could probe the permittivity values with an electric field that was polarized along metal coatings and perpendicular to it, those fits were made separately and allowed for the determination of any anisotropy of the observed permittivity.

The methodology and materials for the experimental data that were used here came from the previously published results, where this information can be found, and it will not be repeated here.

## 3. Results

### 3.1. Bare TFBG Properties

**Figure 1:**
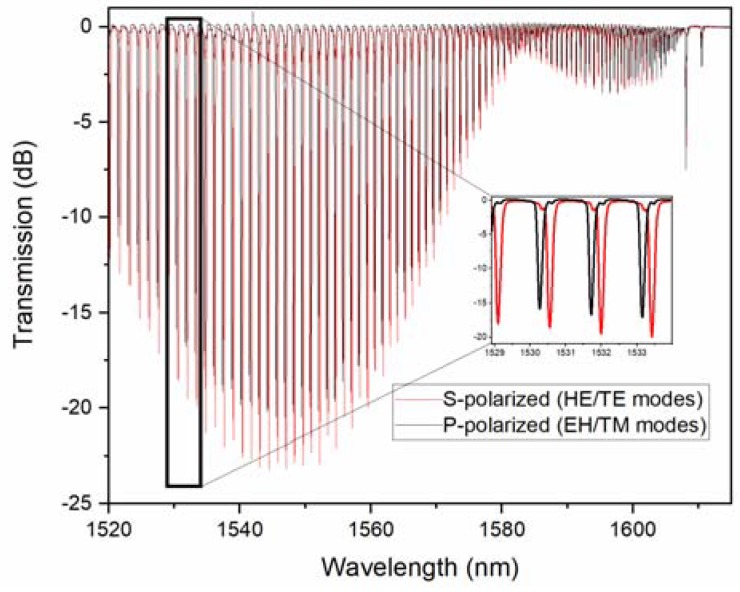
Measured transmission spectrum of a 10° tilt, 10 mm-long tilted fiber Bragg grating (TFBG) in the air for two input states of polarization.

The transmission spectra of a TFBG in air is shown in Figure 1 for input light (core guided) that was polarized parallel to the direction of the tilt (P-polarized) and perpendicular to it (S-polarized).

**Figure 2:**
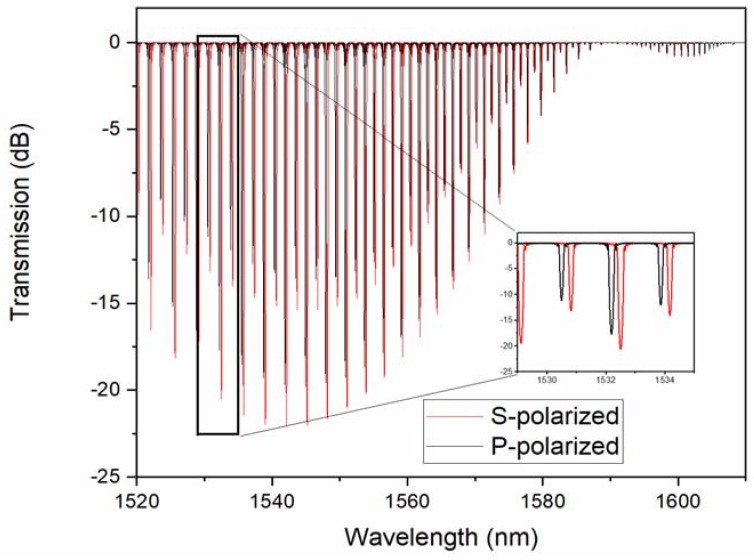
Simulated transmission spectrum of a 10 degree tilt, 10 mm-long TFBG for two input states of polarization.

The difference between the P and S polarized spectra came about for the following reasons. Since the air-cladding interface had a relatively large refractive index difference (0.44 at these wavelengths), the weakly guiding approximation did not apply and the full vector modes needed to be considered. It turned out that the high order cladding modes, (i.e., with effective indices lower than 1.4) separated into two groups that had electric field vectors that were oriented either radially at the cladding boundary (the EH and TM mode families) or tangentially (the HE and TE mode families), and furthermore, that the tilted grating couples P, polarized the input light into EH/TM modes, and S polarized the input couples to HE/TE modes [32]. This property of TFBGs was essential for the sensing application, in which the metal coatings were involved. For instance, the surface plasmon resonance based sensors required TM polarized light at the metal film surfaces, which corresponded to the radially polarized light on the fiber claddings. Without a tilted grating to select the correctly polarized modes, about half of the light that was propagating in a round fiber SPR device had the ‘wrong’ polarization to excite a surface plasmon wave, and thus contributed a background signal that limited the signal to noise ratio. Figure 2 further shows the simulated spectra corresponding to the experimental ones that are shown in Figure 1, indicating that the model that we used to analyze the sensor data was sufficiently accurate to evaluate the effect of the coatings and the surrounding media on the observed spectral changes.

### 3.2. Chemical Vapor Deposition of Copper Coatings

**Figure 3:**
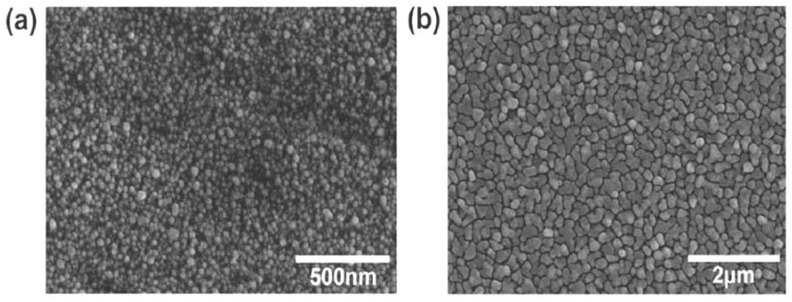
Scanning electron micrographs of copper films on fiber with an average thickness of (**a**) 5 nm and (**b**) 40 nm.

The first example was that of copper that was deposited using a chemical vapor deposition system, as described in [33]. The pulses of a precursor gas of copper guanidinate ([Me_2_NC(^i^PrN)_2_Cu]_2_) were injected into a tube furnace, which was maintained at 200 °C and, under these conditions, the precursor dissociated and condensated as metal on all of the exposed surfaces. A TFBG was inserted in the process chamber to monitor the changes that occurred during the deposition. While each pulse of gas deposited a nominally identical quantity of copper on the surface of the TFBG, the thickness growth occurred through a nucleation process, whereby the initial copper film was highly granular and remained so, because each additional pulse tended to condensate more favorably on the existing copper surfaces than on the exposed glass. Figure 3 shows the typical coatings on glass for thin (5 nm) and thicker (40 nm) films, which confirmed the discontinuous nature of even a relatively thick coating.

**Figure 4:**
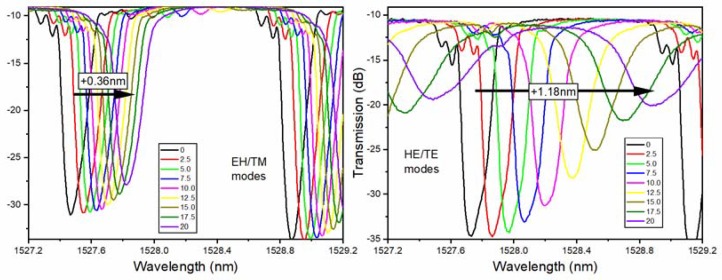
Evolution of P-polarized (**left**) and S-polarized (**right**) mode resonance, as a function of copper thickness in nm (as in figure legend).

For such coatings, the optical properties that were experienced by light polarized in the plane of the surface, would be vastly different than those for the light that was polarized normally to the surface. For the latter, the mobility of the electrons remained limited by the average film thickness, which occupied a very small fraction of the light wavelength for all of the thicknesses below 50 nm. On the other hand, for the light that was polarized in the plane of the film, the mobility of the conduction electrons, and hence of conductivity and optical permittivity, should have depended strongly on the connectivity between the metal particles, seen in Figure 3. Furthermore, this connectivity should have evolved with the number of deposited pulses (and the average thickness accordingly). Figure 4 shows the evolution of the transmission spectrum of the TFBG around a typical resonance of each type, following exposure to an increasing number of gas pulses.

**Figure 5:**
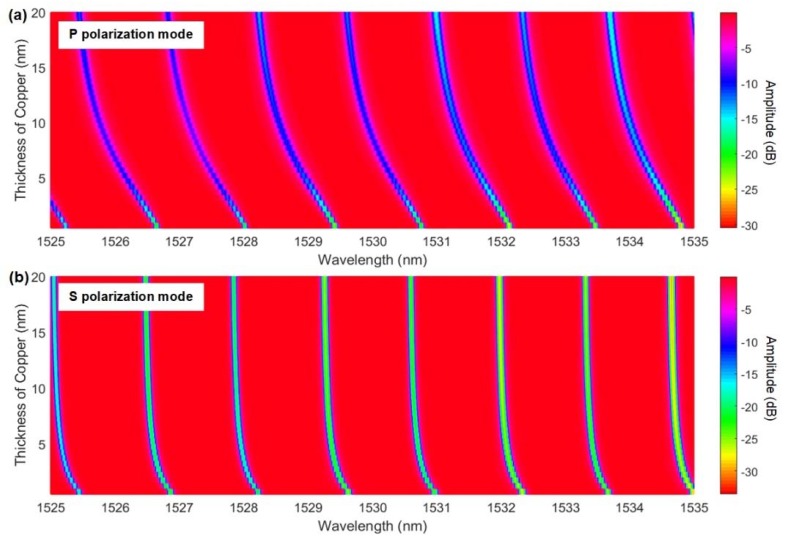
Simulation of the evolution of (**a**) P-polarized and (**b**) S-polarized resonances as a function of copper thickness, using the ‘bulk’ permittivity of copper at these wavelengths.

It was clear that HE/TE modes shifted roughly three times faster than the EH/TM modes for average thicknesses, from 0 to 20 nm, and were always towards the longer wavelengths. Such red shifts of the cladding mode wavelengths were expected when the coatings that were added to an OWG had higher refractive indices than the medium that they were replacing (air here). However, the refractive index of copper at these wavelengths was lower than 1 (0.7 actually [34]) and when we simulated the effect of such copper coatings on TFBG resonances, as shown in Figure 5, all of the resonances shifted towards shorter wavelengths, and by a larger amount for EH/TM modes (by −1.2 nm) than for the HE/TE modes (−0.39 nm).

### 3.3. Chemical Vapor Deposition of Gold Coatings

**Figure 6:**
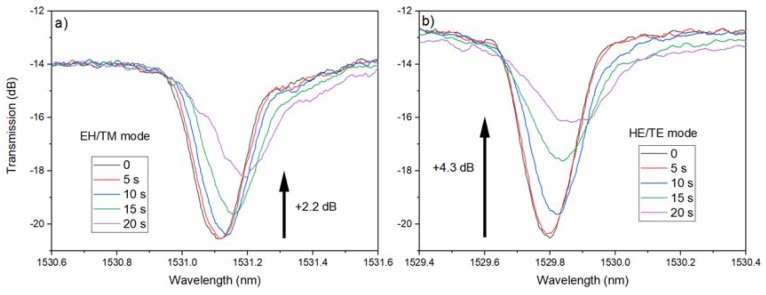
Evolution of (**a**) P-polarized and (**b**) S-polarized cladding mode resonances at various times (indicated in the legend) during the deposition of a 50 nm thick gold coating by CVD.

In this case [11], a finite quantity of a gold precursor compound ([Au(NiPr)_2_CNMe_2_]_2_) was heated to 225 °C in an evacuated chamber, where a TFBG was inserted. Unlike the case of the pulsed chemical vapor deposition (CVD) copper that was previously described, all of the precursor evaporated and dissociated at that temperature and deposition of ‘metallic’ gold on all of the exposed surfaces occurred in a single event. Films of different thicknesses were obtained by changing the amount of the precursor that was introduced in the process chamber. Figure 6 shows the evolution of a pair of EH/TM and HE/TE modes during the first 20 s of deposition, during which the coating grew to an average thickness of 50 nm.

It was not possible to determine exactly the average thickness for each of the times shown in Figure 6, because the deposition proceeded nonlinearly, however it was clear that, similarly to copper, all of the resonances shifted towards longer wavelengths and there were significant differences between the modes, according to their polarization state at the cladding surface, with HE/TE modes showing larger changes than the EH/TM modes.

**Figure 7:**
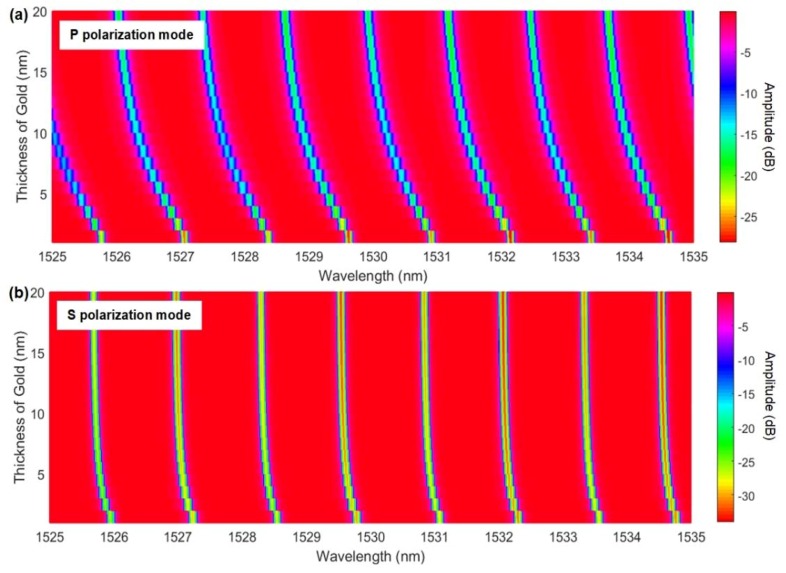
Simulation of the evolution of (**a**) P-polarized and (**b**) S-polarized resonances as a function of gold thickness, using the bulk permittivity of gold at these wavelengths.

Again, simulations with accepted values for gold refractive indices at these wavelengths were carried out, as shown in Figure 7. Similar to the copper case, gold had a bulk refractive index that was lower than one (i.e., a real part of 0.51 [34]), and the simulated resonance all followed the blue shift with the increasing gold thickness (by −1020 pm for EH/TM modes and −245 pm for HE/TE modes at 20 nm of gold thickness).

### 3.4. Electroless Gold Plated Coatings

Another method to deposit metal coatings that generated interest in the fiber sensor community was electroless plating, whereby successive immersions of the TFBG surface in solutions for surface preparation, a gold nanoparticle colloidal solution, and a mixture of chloro-auric acid and hydrogen peroxide in water were used to grow a nanoscale gold film conformally around the fiber surface. The details of the process could be found in [35], but the results that were presented here were new. The example that was chosen here was that of two gold platings that were obtained in nearly identical conditions (same solutions, all at room temperature), but for slightly different plating times (4.6 min for coating A and 7.25 min for coating B), but similar average thickness (27.4 nm vs. 28.9 nm respectively). 

**Figure 8:**
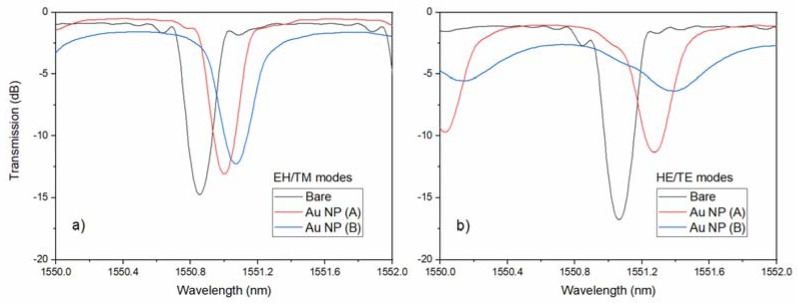
Differences in the (**a**) P-polarized and (**b**) S-polarized cladding mode resonances between a bare TFBG and two others with different gold coatings deposited by electroless plating: coatings A and B have similar thicknesses near 28 nm, but coating A was deposited faster (4.6 vs. 7.25 min).

**Figure 9:**
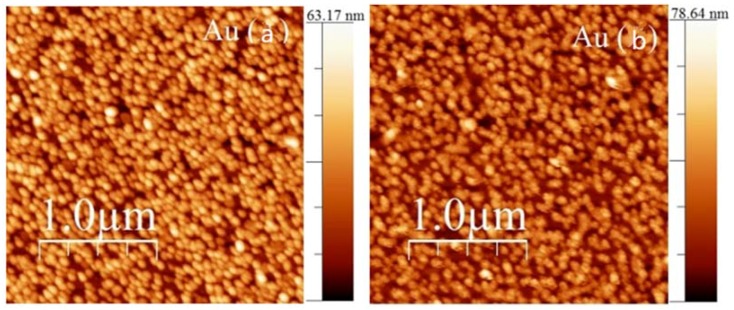
Atomic force microscopy (AFM) images of the surfaces of coatings (**a**) and (**b**), as labeled.

Figure 8 shows how a typical plating process modified the resonances of a TFBG for the two kinds of mode polarization. While EH/TM modes shifted and decreased by relatively small amounts, the tangentially polarized modes (HE/TE) not only shifted more, but by vastly different quantities for films A and B. The morphology of both films, which was obtained by atomic force microscopy, are shown on Figure 9.

### 3.5. Chemical Sensing Enhancements from Thin Metal Coatings

**Figure 10:**
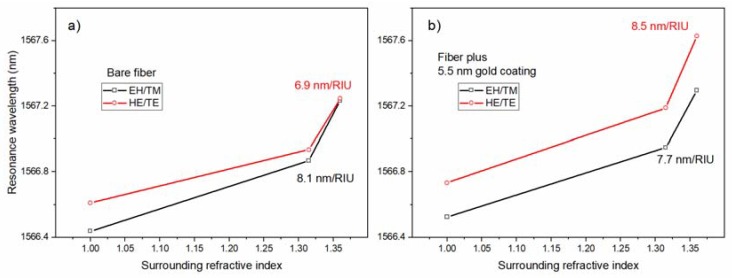
Measured shifts of a pair of cladding mode resonances from a TFBG in air and in two different surrounding media (water and a saline solution), (**a**) for a bare TFBG and (**b**) for a TFBG with 5.5 nm of gold on its surface.

Aside from the impact of the metal/nanoparticle coatings on the optical properties of the TFBG mode resonances, what was actually important was their impact on the sensitivity of these TFBGs when used as chemical sensors. In the first example, an ultrathin gold coating that was obtained by an electron-beam physical deposition system at room temperature, was applied to the surface of a TFBG and was tested for refractometric sensitivity [12]. With an average gold thickness of only 5.5 nm, Figure 10 shows that the response of the HE/TE resonances to the presence of the various surrounding media is significantly different. Normally, the sensitivity of the EH/TM modes to the bulk refractive index changes around the fiber were larger than those for the HE/TE modes, because the former had deeper penetrating evanescent fields, as seen for the bare fiber result, in Figure 10a, where the sensitivity of the HE/TE modes was 15% lower in the important index range between 1.315 (water, at 1550 nm) and 1.36. However, with the addition of a very thin layer of gold nanoparticles, the sensitivity of the HE/TE resonances to the bulk refractive index change became more than 10% larger than for the other polarization (Figure 10b).

**Figure 11:**
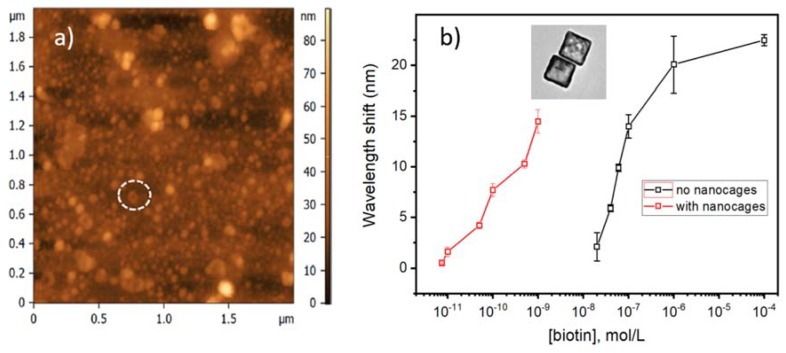
(**a**) AFM image of the surface of a TFBG coated with a monolayer of 10 nm thick gold nanocages; (**b**) Wavelength shifts of the most sensitive resonance of TFBGs functionalized with avidin molecules in the presence of biotin at various concentrations (adapted with permission from [9]. Copyright (2014) Elsevier).

The second example illustrated a slightly different concept, whereby a monolayer of gold nanocages, which were attached on the surface of a TFBG, were used to improve the limit of detection for the proteins in solution [9]. In this case, the unpolarized light was used and the measured responses resulted from the average transmission from the two groups of polarized cladding modes. Figure 11 shows an AFM image of the fiber surface that was coated electrostatically with a 10 nm wide gold nanocages (TEM image of isolated nanocages is shown in the inset of Figure 11b). The biochemical sensing results are shown in Figure 11b, where the presence of the nanocages clearly improved the measured detection limit by more than three orders of magnitude.

## 4. Discussion

**Figure 12:**
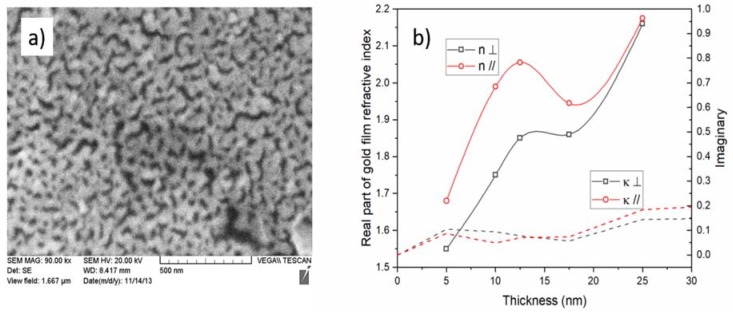
(**a**) Scanning electron micrograph (SEM) image of 20 nm thick gold film deposited by electron beam physical deposition; (**b**) effective parameters of the complex refractive index (n-iκ) of CVD gold coatings as a function of average thickness, determined from the TFBG resonance shifts at wavelengths near 1550 nm (adapted with permission from [38]. Copyright (2014) American Chemical Society).

The findings that were described in the previous sections all pointed to the fact that the thin metal coatings had a strong impact on the propagation properties of the modes of the OWGs on which they were deposited, and that this impact depended on the state of the polarization of the modes at the OWG/metal interface. It was further determined that when such metal coatings were used to enhance the performance of the OWG sensors through plasmonic effects, more polarization-dependent features could be found. However, attempts to model the performance of such metal coated OWG structures failed when the widely accepted values of the metal permittivity were used and when the thickness of the metal layer was smaller than a few tens of nm, which often gave the opposite effects to those that were observed experimentally. This was a consequence of the well-known fact that the ultrathin metal layers had properties that were completely different from those of the bulkier samples, and that the differences depended strongly on the fabrication procedures that were used to form the thin layers [30]. In particular, for the layers thinner than a so-called ‘percolation threshold’, the real part of the permittivity of the metals became positive and their conductivity (and associated optical loss) became very small. This was because such layers were inherently discontinuous, because a thin film growth proceeded through nucleation/condensation, which favored a deposition on the existing metal surfaces instead of on the exposed areas of the substrate, which led to voids and trenches. This can be seen on Figure 12a, which shows a scanning electron micrograph (SEM) of the surface of a fiber that has been coated with a 20 nm thick film of gold deposited by evaporation (driven by an electron beam heater).

In such a film, the motion of the conduction electrons, which were driven by an electric field that was polarized perpendicularly to the film, was limited by the film thickness to the values that were well below the mean free path of the electrons in the bulk metal (37.7 nm for gold and 39.9 nm for copper, for instance [31]), from which the complex permittivity was calculated. Therefore, the effective permittivity that was perceived by the EH/TM modes began to change from the bulk value, as the coating thicknesses decreased under a few tens of nm. For the light that was polarized parallel to the layer surface however (i.e., the evanescent field of the HE/TE modes), the motion of the conduction electrons was restricted by the connectivity of the material that formed the layer as well as the AFM and SEM images that have been presented in this paper, for the thin metal films that were obtained by several processes showed that the connectivity could vary greatly. For the films with a high connectivity (few voids and trenches), the conductivity of even the very thin films (a few nm) could approach that of the bulk, because the conduction electrons were driven in the plane of the film. However, in layers with poor conductivity, the permittivity then changed rapidly away from the bulk. In order to investigate this, we used inverse modelling to estimate the effective complex refractive index of the thin gold layers from the resonance shifts of the polarized TFBG modes, on which those layers were deposited. The results are shown in Figure 12b for the gold layers, which ranged in thickness from 5 to 25 nm. Since the bulk value of the complex refractive index of gold at wavelengths near 1550 nm was 0.52-i10.7, it was clear that here, the ‘metal’ behaved as a very good dielectric (high real part of the refractive index and low imaginary part). This was because such thin films were below the ‘percolation threshold’, whereby the connectivity increased to the point where the average mean free path of the conduction electrons in discontinuous metal films reached the mean free path of the bulk [13,36,37]. Furthermore, the results pointed to a huge birefringence of these thin gold films, which explained the vastly different behaviors that were reported in this paper for the guided modes with different polarization states, which included a strong impact on the sensing performance.

## 5. Conclusions

The purpose of this paper is to highlight the fundamentally different nature of the very thin metal coatings that are used to enhance the performance of optical sensors and the impossibility of using the literature values for their optical properties when designing or analyzing them. Furthermore, the high differential sensitivity of the HE/TE modes, relative to the EH/TM modes, to the presence of those thin metal films appears to indicate that the highly sensitive devices with low limits of detection could be developed by exploiting those differences. While a TFBG platform was used here to demonstrate these properties of the thin metal coatings that are used in sensing, the conclusions are general and apply to other sensor systems, including long period gratings, large core/cladding removed fiber SPR sensors, D-shaped fibers, and tapered fibers [39]. The only advantages of TFBGs over these other platforms are the inherent referencing by the core mode Bragg resonance and the possibility of the polarization control of the probe field at the fiber surface.
